# Physiological responses and morphological adjustments in provenances of two woodland species in a chronic drought experiment

**DOI:** 10.1093/treephys/tpag117

**Published:** 2026-08-22

**Authors:** Rhys Browning, Stefan K Arndt

**Affiliations:** School of Agriculture, Food and Ecosystem Sciences, Faculty of Science, Burnley Campus, The University of Melbourne, 500 Yarra Boulevard, Richmond, Melbourne, Victoria, 3121, Australia; School of Agriculture, Food and Ecosystem Sciences, Faculty of Science, Burnley Campus, The University of Melbourne, 500 Yarra Boulevard, Richmond, Melbourne, Victoria, 3121, Australia

**Keywords:** *Eucalyptus*, intraspecific variation, osmotic potential, plasticity, rainfall exclusion, stomatal conductance, water potential

## Abstract

Drought-induced tree dieback is impacting forest and woodland ecosystems. One approach to mitigate this is to plant better adapted genotypes of species from warmer and drier climates. This requires understanding drought adaptation both interspecifically and intraspecifically, which we investigated in this common garden experiment. Six genotypes from two related *Eucalyptus* species, *Eucalyptus microcarpa* Maiden and *Eucalyptus melliodora* A.Cunn. ex Schauer, were selected from geographically disjunct locations, five of which encompass the drier ranges of each species. One-year-old saplings were planted in soil under a rainout shelter. After a 5-month adjustment period, irrigation was withheld for 17 weeks to emulate drought, while control trees were irrigated twice weekly. Predawn (Ψ_pd_) and midday (Ψ_md_) water potentials and stomatal conductance (g_s_) was measured weekly. Trees were harvested at the end of the drought period, and biomass and leaf osmotic potential were measured. We observed significant differences between species, and only limited evidence for intraspecific variation. Neither species developed substantial water deficits during the drought, but both species exhibited osmotic adjustment. Ψ_pd_ and Ψ_md_ were significantly lower and g_s_ was higher in droughted *E. microcarpa* in comparison with droughted *E. melliodora*, however the differences were small. Decreases relative to control trees in biomass were greater in *E. microcarpa* (57%) than *E. melliodora* (41%). Root:shoot ratios in droughted trees were comparatively greater than well-watered trees in both species, however, dry root weight was greater in *E. melliodora* across both treatments. Only *E. microcarpa* had significantly lower specific leaf area (10%) and tree height (26%) in comparison with well-watered trees. Provenances of *E. microcarpa* from drier regions exhibited less negative Ψ_pd_, less osmotic adjustment and comparatively lower height in droughted vs well-watered trees. Our results highlight the complexity of local drought adaptation and that morphological changes may be as important as physiological trait responses for drought resistance.

## Introduction

Many forest and woodland ecosystems are being adversely affected by altered temperature and precipitation patterns from climate change (Dey et al. 2019), which can significantly affect landscape composition through drought-induced tree mortality (Breshears et al. 2005, Ellison et al. 2005). These impacts are further exacerbated by the extent of landscape fragmentation in many of these ecosystems (Saunders et al. 1991, Prober et al. 2013). Approaches of forest restoration that seek to maintain the current species distributions need to be evaluated. One approach involves ‘provenancing’, which seeks to utilize intraspecific variation and localized adaptation of tree populations from varying climates within the natural distribution of a given species. In the context of climate change and drought stress, this involves utilizing seed from populations originating from hot and/or dry climates, with the assumption that these populations are adapted to their respective climates (Hoffmann et al. 2021). These adapted genotypes are then planted with the intention of establishing landscapes that are more resilient to future droughts (Prober et al. 2015). To optimize the potential for provenancing, it is important to understand population-level adaptation within a species, which in turn may allow for the targeted selection of drought-adapted genotypes in land restoration (Axelsson et al. 2020).

Drought will impact tree species in various ways. However, when water availability to tree roots is limited, and if transpiration demands in the canopy remain, trees are susceptible to xylem cavitation which can lead to tree mortality (Choat et al. 2018). To prevent xylem cavitation, trees have evolved a suite of co-ordinated traits in response to water limitation. Many species conserve water via tighter regulation of stomata or shedding of leaves, which avoids substantially negative water potentials (Ψ) (Tardieu et al. 1993). This reduction in transpiration reduces the risk of cavitation as xylem vessels can embolize as Ψ decreases (Meinzer et al. 2016). Other species can also avoid negative Ψ by accessing deeper soil moisture through extensive root systems (Nolan et al. 2018), increasing root:shoot ratio (Nolan et al. 2017) or primarily occupying riparian environments. In contrast, other species can tolerate water deficits, often demonstrating less strict control of stomata, and can therefore continue to transpire at lower Ψ (Tardieu et al. 1993). Various adaptations allow for continued function at lower water potentials, including a more negative water potential at turgor loss point (Ψ_tlp_) and greater xylem resistance to cavitation (Choat et al. 2018). This may result in increased growth and competitiveness in medium-term droughts (Sade et al. 2012).

There are over 800 *Eucalyptus* species globally that occur in a vast variety of ecosystems and climates (Brooker 2000). Traits associated with a heightened tolerance to water deficits can vary with the climate in which *Eucalyptus* species occur. *Eucalyptus* species that occur in more arid climates often have a more negative turgor loss point (Bourne et al. 2017, Li et al. 2018), greater osmotic adjustment (Merchant et al. 2007), narrower xylem vessels (Pfautsch et al. 2016) and increased xylem resistance to embolism (Oliveira et al. 2022). Furthermore, eucalypts demonstrate varying levels of plasticity in response to drought, including adjustment of leaf morphology (Mclean et al. 2014), specific leaf area (SLA) (Steane et al. 2017) and sapwood area to leaf area (LA) ratio (Huber value, H_V_) (Carter and White 2009, Pritzkow et al. 2020*a*).

An increase in tolerance to water deficits appears to correlate with aridity of origin across many *Eucalyptus* species (Salvi et al. 2022), though there are exceptions to the rule. For example, the phreatophyte *Eucalyptus camaldulensis* is able to persist in very arid climates through stringent stomatal regulation and comparatively greater root:shoot ratios allowing access to water in regions with relatively low annual rainfall (Nolan et al. 2017, 2018). It can therefore be problematic to draw conclusions on drought adaptation through trait or climate niche analysis only.

We have a relatively limited understanding of the variability of functional traits related to drought within species in the *Eucalyptus* genus. However, there are recent studies that have investigated the intraspecific variation of physiological traits related to drought adaptation (Lucani et al. 2019) or leaf structural traits and Huber value (Anderegg et al. 2021, Mclean et al. 2014, Pritzkow et al. 2020*b*). These studies indicate that, in certain species, *Eucalyptus* genotypes from more arid regions may exhibit differences in both fixed and plastic functional traits. However, whether the observed intraspecific variation in trait differences results in different growth rates and/or drought survival is yet to be determined. Furthermore, studies that investigated intraspecific variation of drought responses are often pot studies on <1-year-old saplings (Gibson et al. 1995, Li 1998, Li et al. 2000). While pot studies enable this logistically challenging research, they can impact results, especially through root restriction (Brunner et al. 2015, Kunz et al. 2016).

Here, we present a common garden drought study comparing water use and drought responses of six provenances of each of two eucalypt species, *Eucalyptus microcarpa* Maiden and *E. melliodora* A.Cunn. ex Schauer, subjected to a chronic, in-ground drought experiment. While the species occupy similar woodland ecosystems and have overlapping climate niches, *E. microcarpa* extends further inland into regions with lower rainfall and higher aridity. The populations of each species were sourced from regions that differ in both yearly and seasonal precipitation and temperatures. We hypothesized that *E. microcarpa* would exhibit greater tolerance to drought via a maintenance of stomatal conductance and continued function at lower water potentials, resulting in greater growth and survival. We also hypothesized that localized adaptation in provenances within each species would result in genotypes from more arid regions also exhibiting greater tolerance to water deficits and would subsequently have greater growth.

## Materials and methods

### Species selection

*Eucalyptus microcarpa* and *Eucalyptus melliodora*, commonly referred to as Grey Box and Yellow Box respectively, are broadly distributed eucalypt species that form integral components of woodland habitats in south-eastern Australia. Climate modelling suggests these woodland regions will be disproportionately affected by drought in the coming decades (Metcalfe and Bui 2017). Both species reside in subgenus *Symphyomyrtus*, section *Adnataria* (boxes and ironbarks) (Nicolle 2024). The distributions are overlapping, but *E. microcarpa* extends further inland, encompassing regions with lower rainfall than *E. melliodora* (Figure 1, Table 1). Populations of both species in south-eastern Australia have been affected by drought-induced dieback in recent years, prompting Bush Heritage Australia (BHA) to conduct a provenance revegetation trial in the Nardoo Hills Conservation Reserve in northcentral Victoria. In 2019, five provenances of each species that span a climatic gradient of temperature, rainfall and rainfall seasonality were planted in a randomized block trial. This was intended to integrate drought-adapted genotypes of these two keystone tree species into a regenerated woodland ecosystem. Since establishment, growth and survivorship has undergone regular monitoring (McDonald et al. 2021).

**Figure 1:**
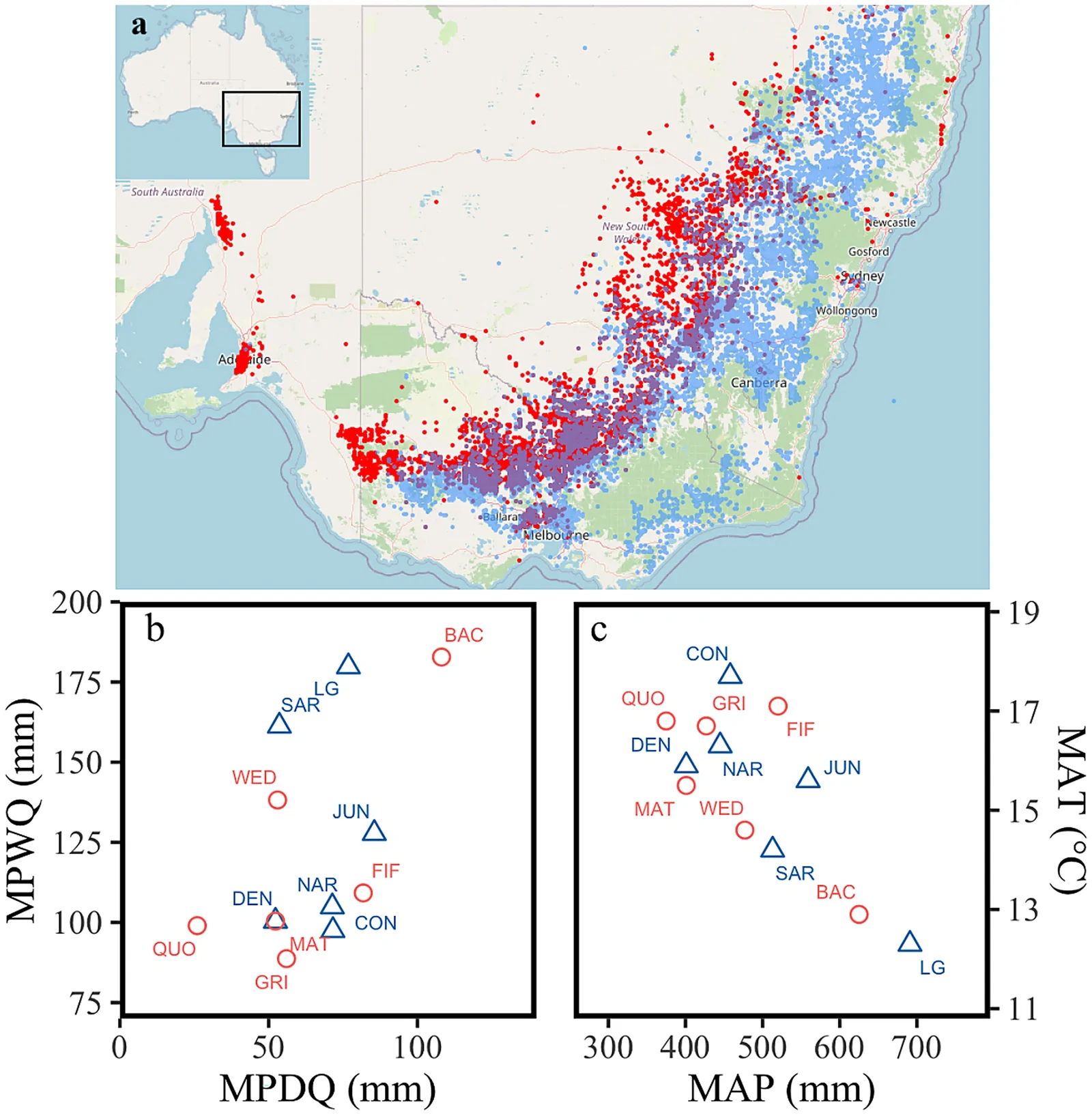
(a) Distribution of *E. microcarpa* (red) and *E. melliodora* (blue) in south-eastern Australia extracted from the atlas of living Australia (www.ala.org.au). Regions where both species occur are coloured purple. (b) Seasonal precipitation for each provenance of *E. microcarpa* (red circles) and *E. melliodora* (blue triangles). Median precipitation in the driest quarter (MPDQ) is on the x-axis and MPWQ on the y-axis. (c) Mean annual precipitation (MAP) and mean annual temperature (MAT). Provenance codes can be referenced from Table 1. MPDQ and MPWQ was sourced from Atlas of Living Australia. MAP and MAT data were sourced from bom.gov.au. Weather station information is available in the supplementary material (Table S1 available as Supplementary data at *Tree Physiology* Online). The map was sourced from OpenStreetMap (openstreetmap.org/copyright). Distribution data sourced from Atlas of Living Australia https://doi.org/10.26197/ala.a05303a9-4651-48ad-a6d8-881ec0b26ef7.

**Table 1:** Precipitation and temperature for each population. The local populations and populations not included in the Bush Heritage Australia trial are noted. Data were collected from the Bureau of Meteorology (BOM) Australia, except for species mean annual temperature and mean annual precipitation, which was determined to be the mean value of the distribution collected from the atlas of living Australia (www.ala.org.au). Weather station information is available in the supplementary material (Table S1 available as Supplementary data at *Tree Physiology* Online).

	MPDQ	MPWQ	MAT	MAP
	Median precipitation driest quarter (mm)	Median precipitation wettest quarter (mm)	Mean annual temperature (°C)	Mean annual precipitation (mm)
***Eucalyptus microcarpa***				549
(BAC) Bacchus Marsh ^(Not included in BHA Trial)^	108	183	12.9	625
(FIF) Fifield	82	109	17.1	520
(GRI) Griffith	56	89	16.7	427
(MAT) Mathoura	52	100	15.5	401
(QUO) Quorn	24	83	16.8	375
(WED) Wedderburn ^(BHA Local Population)^	53	111	14.6	477
***Eucalyptus melliodora***				687
(CON) Condobolin	72	96	17.7	458
(DEN) Deniliquin	52	100	15.9	401
(JUN) Junee	86	128	15.6	559
(LG) Langhi Ghiran ^(Not included in BHA Trial)^	77	180	12.3	691
(NAR) Narrandera	71	105	16.3	445
(SAR) St Arnaud ^(BHA Local Population)^	50	111	14.2	513

We used the same seed that was utilized in the provenance trial at Nardoo Hills for this experiment. To complement the common garden revegetation experiment, we established a common garden, chronic drought experiment to allow a more accurate determination of physiological responses to drought. Furthermore, to further our understanding of intraspecific variation in drought responses, we extended the climatic gradient via an additional provenance of each species (Table 1).

### Experimental design

This study was conducted at the University of Melbourne, Burnley Campus, Melbourne (Australia) (−37.830, 145.023). Seed from six populations of each species was germinated in a glasshouse in July 2021 on a seed raising mix consisting of 80% medium pine bark, 10% sieved coir peat and 10% washed coarse sand with 1.5 kg SaturAid (Evergreen Garden Care Australia) and 1 kg of dolomite per m^3^. In January 2022, 15–20 saplings from each provenance were then re-potted in 150 mm plastic pots in native seed raising mix consisting of 85% medium pine bark and 15% washed coarse sand, 4 kg Macracote Colonize Plus 18 N:1P:10 K (plus trace elements) (Langley Fertilisers, Australia), 1.5 kg SaturAid and 1 kg dolomite lime per m^3^. Plants were then placed outside with overhead irrigation until planting.

Eight plots of 7 m × 4.5 m were established under a polythene rainout shelter with open sides to allow for ventilation. The site is on an alluvial flood plain, consisting of a topsoil of sandy loam and a sub-soil of sandy-clay loam at roughly 1 m. A waterproof root barrier (Permathene Australia Pty Ltd) was inserted in 1 m trenches along the perimeter of each plot. Overhead irrigation was installed above each plot to imitate natural rainfall. Trees were planted in the ground in October 2022 and allowed to establish for a 5-month period. Two replicate trees per provenance for each species were planted in each of the eight blocks (2 trees × 6 provenances × 2 species = 24 trees per block), resulting in eight replicates per provenance per treatment. However, due to increased edge effects, plants in the first and last row were removed from the experiment. This resulted in five to six plants per provenance per treatment. The experiment began in February 2023 when irrigation was stopped in the four treatment blocks while the control blocks were watered twice weekly for 17 weeks. The control trees were irrigated at ~4.4 L m^2^, which would emulate 4.4 mm of rain over a period of 20 min during each irrigation day.

### Weather monitoring

An Atmos-41 weather station attached to a ZL6 logger (METER Group, Inc., WA, USA) was installed in the centre of the rainout shelter. Temperature and humidity were measured at 5-min intervals. To determine vapour pressure deficit (VPD), the saturated vapour pressure was first calculated via the following equation as per Allen (1998):


$$SVP=0.6108\cdotp \exp \left(\frac{17.27\cdotp T\ }{T+237}\right)$$

where T is temperature in degrees Celsius. VPD (kPa) was then calculated via the following equation as per (Allen 1998):


$$VPD= SVP\left(1-\frac{RH\ }{100}\right)$$

where RH is the relative humidity. Daily mean VPD on measurement days was then calculated by averaging VPD between the hours of 10.00 and 17.00 h.

### Leaf water potential and leaf gas exchange

Leaf water potential was measured weekly (except week 16) for the duration of the experiment. On each measurement day, pre-dawn water potentials (Ψ_pd_) were measured between 04.30 and 05.30 h and mid-day water potentials (Ψ_md_) measured between 13.00 and 14.00 h. One fully expanded leaf was selected from the mid-canopy of the north facing side of each plant. Measurements followed the protocol outlined in (Rodriguez-Dominguez et al. 2022). Briefly, sampled leaves were placed in a sealed plastic bag with the air removed and kept in a dark, cool container until measurements occurred (no longer than 1 h after sampling). Measurements were conducted with a Scholander-type pressure chamber (Scholander et al. 1965) (Soilmoisture Plant Water Console 3000, Goleta CA, USA). Stomatal conductance was measured using an LI-600 handheld porometer/fluorometer (LI-COR Inc., Lincoln, NE, USA). A fully expanded leaf on each tree was measured from the mid canopy on the north side of the tree between 10.00 and 11.00 h and again after Ψ_md_ measurements, between 14.30 and 15.30 h.

### Osmotic adjustment and apoplastic water fraction dilution estimation

To enable a greater number of measurements of osmotic potential (π), freezing point osmometry of expressed sap (π_es_) was used (Callister et al. 2006). However, apoplastic water fraction (AWF) dilution of the expressed sap can affect accurate predictions of leaf osmotic potential calculated from *π_es_* (Arndt et al. 2015). Therefore, a subsample of trees that had osmotic potential measured via osmometry were also measured via pressure–volume curve analysis (π_PV_). A linear model comparing π_PV_ and π_es_ was then developed for each species, and the relationship was used to estimate osmotic potential (π) from π_es_. Leaf sampling took place in week 17. For the measurement of π_es_, two fully expanded leaves per tree were collected before dawn and rehydrated with deionized water in complete darkness. Leaf Ψ was measured with a Scholander-type pressure chamber and leaves were considered fully rehydrated at values above −0.1 MPa. Leaves were pooled for each tree, and leaf discs were then cut from each leaf by avoiding major veins using an 8 mm brass cork borer and frozen in an Eppendorf tube at −20 °C. Upon thawing, leaf discs were centrifuged, and sap from each tree was measured using a calibrated Osmomat 030 freezing point osmometer (Gonotech, Germany) to determine the osmolarity of expressed sap. Osmometer readings were regularly checked for accuracy using known standard solutions of 100 and 1000 mOsmol/kg H_2_O. π_es_ was calculated from the Van’t Hoff equation:


$${\pi}_{es}= RT\bullet{C}_{ES}$$

where R is the gas constant (8.31447 J K^−1^ mol^−1^), T is room temperature (293 K) and C_ES_ is the molar concentration of osmolytes in expressed sap. Leaves used for pressure–volume curve analysis were also collected in week 17. Three leaves per provenance per treatment were measured using the bench dehydration technique (Arndt et al. 2015). Briefly, leaves were collected in the morning and immediately placed in tubes with deionized water and allowed to rehydrate in complete darkness. Following complete rehydration (see above) leaf Ѱ and leaf weight were measured while the leaves dried out on a lab bench. The P–V graph was then analysed based on the model developed by Schulte and Hinckley (1985) and water potential at turgor loss point (Ψ_tlp_) and osmotic potential at full turgor (π_PV_) estimated. Osmotic adjustment (π_adj_) was determined as the difference in π between well-watered and droughted trees, paired at both the species level and the provenance level.

### Huber value and SLA determination

During the drought experiment, two branches per tree were marked with flagging tape on the north-east facing side of the plant. This was to ensure leaf sampling for Ψ measurements did not occur on these branches and impact H_V_ measurements. Immediately after the conclusion of the drought trial in week 17, the two marked branches were removed from between four and six plants per provenance per treatment to determine the sapwood area to LA ratio (Huber value, H_V_). The branches were cut at the main stem and small cross sections of the branch area were taken and photographed under a microscope and measured in ImageJ (version 2.14.0, ImageJ). The xylem diameter and pith diameter were both measured, and the pith area removed from the total xylem area measurement. The leaves of the branches were stripped, and leaves were imaged using a desktop scanner. Total LA was also measured in ImageJ. Huber value (Hv, cm^2^ m^−2^) was determined by the equation:


$$Hv=\frac{Xylem\ area\left({cm}^2\right)\ }{One- sided\ leaf\ area\ \left({m}^2\right)}$$

The SLA (cm^2^ g^−1^) was measured on the same branches. Leaves were dried at 80 °C until reaching constant weight. The SLA was then determined via:


$$SLA=\frac{One- sided\ leaf\ area\ \left({cm}^2\right)}{Leaf\ weight\ (g)}$$

### Biomass determination

Trees were destructively harvested after the conclusion of the drought trial. Trees were first measured for height and then cut at ground level and separated into branches and main stem. The fresh weight of the main stem and the branches was recorded, and the main stem was then dried at 80 °C until reaching constant weight. Fresh branches were then subsampled and measured for LA, stem:leaf ratio, stem dry weight and leaf dry weight. From this, a ratio of stem fresh weight to stem dry weight was used to estimate total dry weight of branches. Leaf area was also estimated from the subsamples to estimate total LA and leaf weight of the harvested trees. Roots were then excavated with an 1.8 ton excavator. An area 60 cm from the stem, and 60 cm deep was excavated and soil was removed from the coarse root material. Roots were then cleaned and dried at 80 °C until reaching constant weight.

### Data analysis

Statistical analyses were performed using R (Version 4.3.2) (R Core Team 2023). Data was visually checked for normality by using q-q plots and homogeneity of variance was checked using Levene’s tests. Breakpoints were used to ascertain the divergence of treatment and control effects on Ψ_pd_ using the strucchange package (Zeileis et al. 2001). Trends in Ψ_pd_, Ψ_md_ and g_s_ were analysed via generalized additive mixed models (GAMMs) using the mgcv package in R (Wood 2011). g_s_ was log-transformed to address positive skewness. Ψ_pd_ was modelled as a function of week only, however Ψ_md_ and g_s_ responses were modelled as a function of both week and VPD. Smooths of week only, VPD only and a combination of week and VPD were used. Model comparison was then completed to ascertain the best fit via AIC. In each model of Ψ_pd_, Ψ_md_ and g_s_, a similar hierarchical modelling approach was used. We first examined treatment effects within each species using treatment as a two-level parametric factor. To then compare species responses to drought, species was used as a two-level parametric factor resulting in separate smooths. A species-specific model was then compared with a null, shared-smooth model via an AIC test. Finally, to examine provenance responses within each species under drought, the same approach was used, however provenance was used as the grouping factor. In all models, individual tree was used as a random intercept to account for repeated measures. Models were fitted using restricted maximum likelihood (REML) and maximum likelihood (ML) for model comparisons. Predicted responses and confidence intervals were extracted from REML models. Paired t-tests were used to compare Ψ_pd_, Ψ_md_ and g_s_ means over the course of the drought experiment. Repeat measures analysis of variance (ANOVA) and TukeyHSD post-hoc tests were performed to assess species and provenance variations in biomass allocation, osmotic adjustment and SLA. Linear regression models were also employed to explore relationships between biomass allocation and traits with climate variables. For all linear models, we assessed the goodness-of-fit using R^2^ values and examined the statistical significance of predictor variables through *p*-values.

## Results

### Effect of drought on leaf water potential and stomatal conductance

Drought treatment significantly reduced Ψ_pd_ in both *E. microcarpa* and *E. melliodora* (Figure 2, Table 2), however neither species exhibited Ψ that approached Ψ_tlp_. The lowest individual reading of Ψ_pd_ in this experiment was −2.36 MPa observed in *E. microcarpa* in week 17 (Data not shown). Species had different trajectories of Ψ_pd_ decline as confirmed via a GAMM model comparison (ΔAIC = 7.7) (Table S3 available as Supplementary data at *Tree Physiology* Online). A significant breakpoint between droughted and well-watered trees occurred at week 9 in both species (sup.F = 83.08, *P* < 0.001). Paired t-tests comparing mean Ψ_pd_ when treatment effects were significant from weeks 10 to 17 was significantly lower in *E. microcarpa* in comparison with *E. melliodora* (Table 3). *E. microcarpa* provenances had small yet significant differences in Ψ_pd_ trajectories under drought (Table S4 available as Supplementary data at *Tree Physiology* Online), suggesting there may be differential rates of decline among provenances. Provenances within *E. melliodora* did not differ.

**Figure 2:**
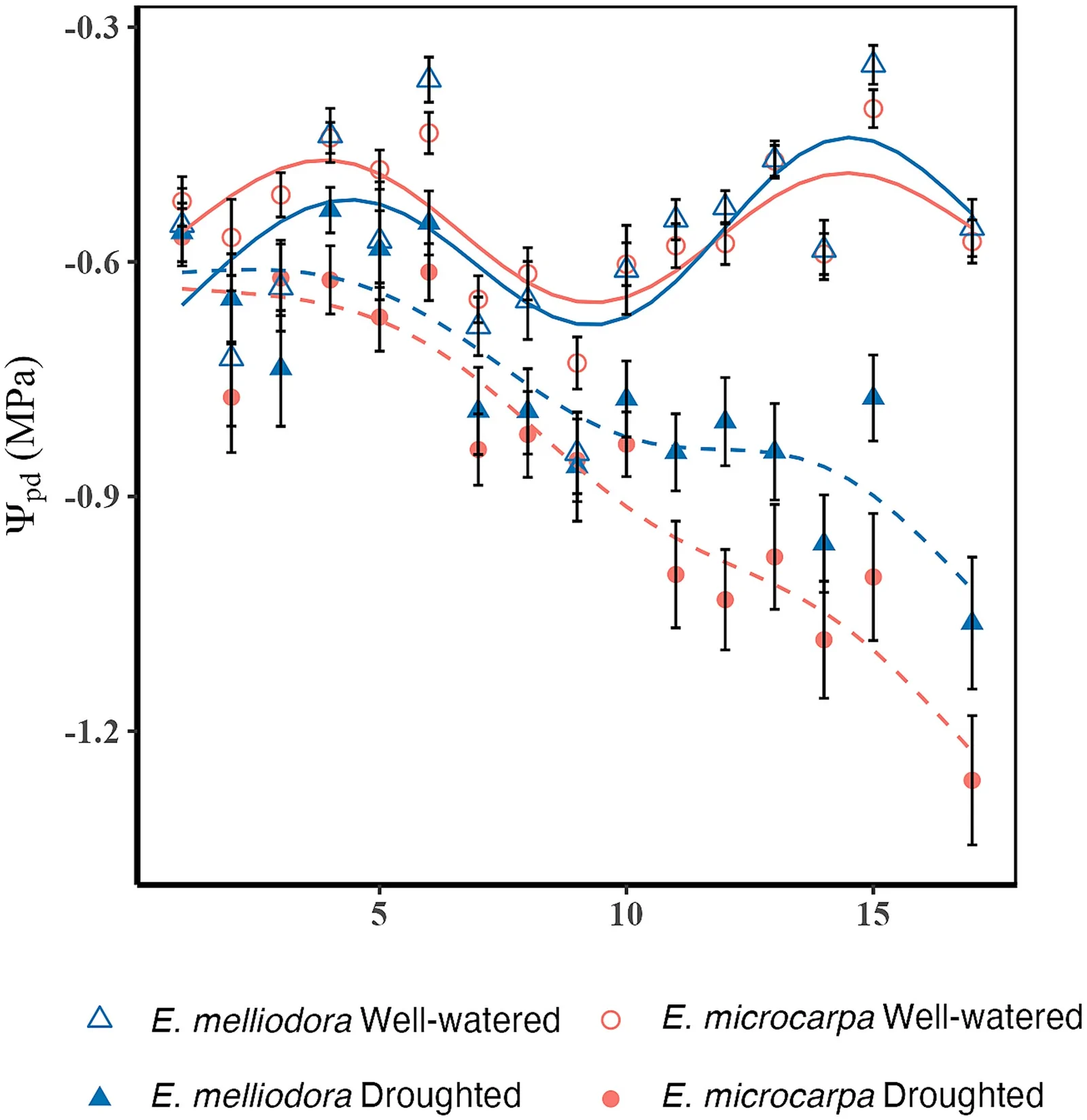
Predawn water potentials (Ψ_pd_) for *E. microcarpa* (red circles) and *E. melliodora* (blue triangles) across 17 weeks of the experiment. Values are mean species values calculated by averaging all provenances in each measurement week (measurement did not occur on week 16). Treatment (droughted) means are represented by closed symbols, control (well-watered) means are represented by open symbols with error bars (± SE). Lines are predicted fits of GAMM models for each treatment × species combination. Solid lines indicate well-watered trees and dashed lines indicate droughted trees.

Drought treatment significantly reduced Ψ_md_ in both species, however the reduction was greater in *E. microcarpa* than *E. melliodora* (Table 2, Figure 3a and c). Overall, Ψ_md_ was highly variable and days of more negative Ψ_md_ values coincided with high atmospheric VPD values (Figure 3c). Mean Ψ_md_ after the break point (Week 10 to 17) for droughted *E. microcarpa* trees was significantly lower than *E. melliodora* (Table 3). Both week and VPD were significant predictors of Ψ_md_ (Table S2 available as Supplementary data at *Tree Physiology* Online) and the responses to week and VPD were similar between species. Introducing provenance into the Ψ_md_ response models did not improve the model fit for either species (Table S3 available as Supplementary data at *Tree Physiology* Online).

**Table 2:** Treatment effects from GAMM models on Ψ_pd_, Ψ_md_ and g_s_ for each species. Treatment effect represents mean difference of droughted trees to well-watered trees. *P*-values are from the parametric term of each GAMM.

Response	Species	Treatment effect	SE	*P*	Provenance difference
Ψ_pd_ (MPa)	*E. melliodora*	−0.190	0.049	<0.001	No (ΔAIC = 28.74)
Ψ_pd_ (MPa)	*E. microcarpa*	−0.301	0.047	<0.001	Yes (ΔAIC = 5.15)
Ψ_md_ (MPa)	*E. melliodora*	−0.335	0.049	<0.001	No (ΔAIC = 226.1)
Ψ_md_ (MPa)	*E. microcarpa*	−0.491	0.061	<0.001	No (ΔAIC = 185.2)
g_s_ (mol m^−2^ s^−1^)	*E. melliodora*	−0.478	−	<0.001	No (ΔAIC = 48.2)
g_s_ (mol m^−2^ s^−1^)	*E. microcarpa*	−0.451	−	<0.001	No (ΔAIC = 29.9)

**Table 3:** T-tests of pooled species mean values of the treatment group from week 10 to week 17 for Ψ measurements, and week 10 to week 15 for g_s_ measurements.

Response	*E. melliodora*	*E. microcarpa*	t	df	*P*
Ψ_pd_ (MPa)	−0.867	−1.03	−6.82	6	<0.001
Ψ_md_ (MPa)	−1.87	−2.15	−13.42	6	<0.001
g_s_ (mol m^−2^ s^−1^)	0.053	0.067	−7.23	5	<0.001

**Figure 3:**
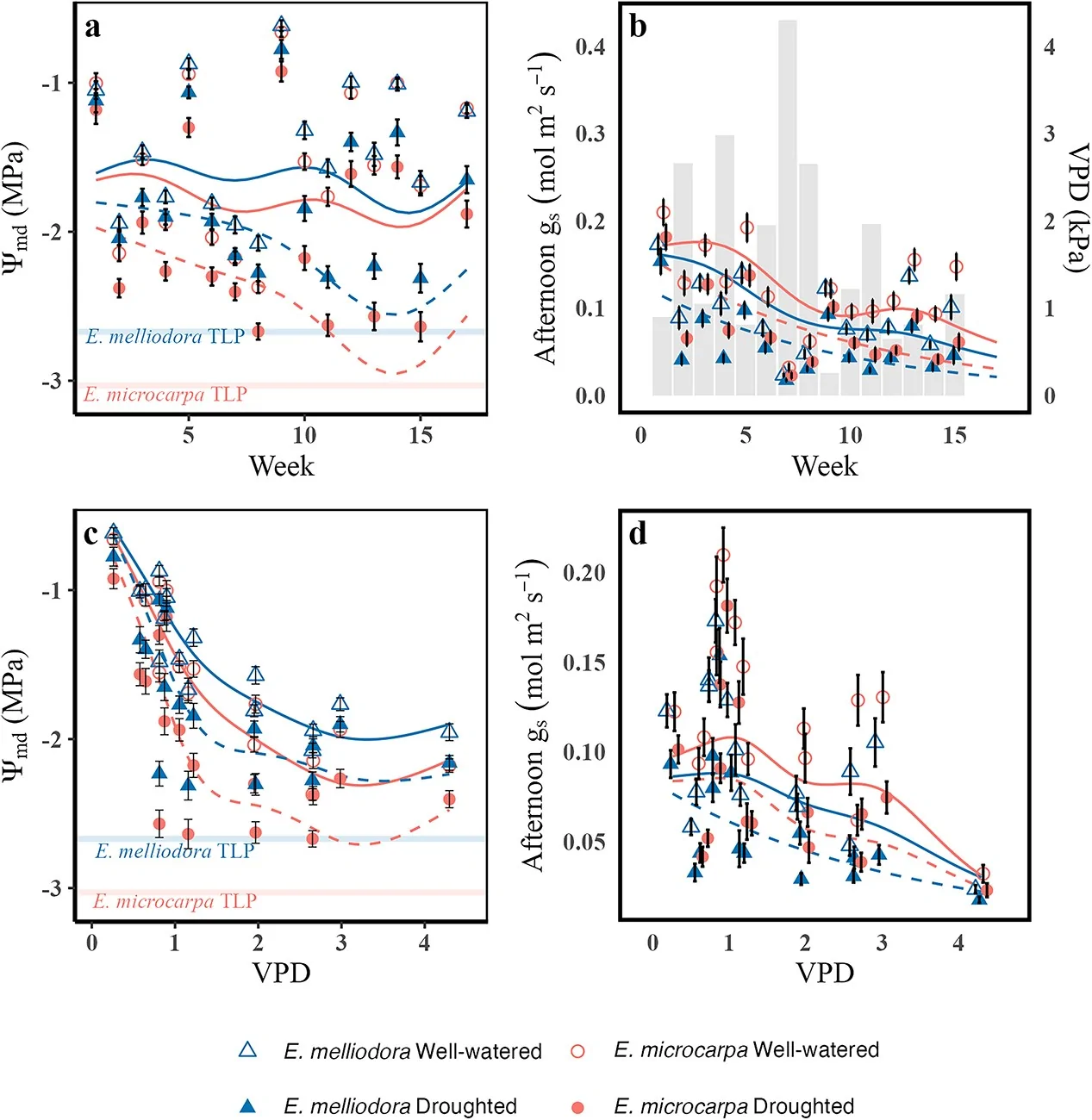
(a) Mean weekly midday water potentials (Ψ_md_) and (b) stomatal conductance (g_s_) over the course of the experiment and (c) Ψ_md_ and (d) g_s_ responses to VPD. Symbols are *E. microcarpa* (red circles) and *E. melliodora* (blue triangles). Treatment (droughted) means are shown as closed symbols, control means as open symbols. Black vertical bars are error bars (± SE). Values are mean species values calculated by averaging all provenances in each measurement week (measurement did not occur on week 16 for Ψ_md_ and week 16 and 17 for g_s_). Lines indicate GAMM models for well-watered (solid) and droughted (dashed) treatments. The weekly trajectories are based off predicted values generated by holding VPD at the observed mean, and VPD responses were generated by holding week at the observed mean. The grey bars in (b) represent mean VPD values (between 08:00 and 17:00 h) for the coinciding measurement days.

Stomatal conductance (g_s_) was significantly lower in droughted *E. microcarpa* (a difference of 0.040 mol m^2^ m^−1^ SE = 0.060, *P* < 0.001) and *E. melliodora* (0.032 mol m^2^ m^−1^ SE = 0.045, *P* < 0.001) (Figure 3b). GAMM models of droughted g_s_ responses to week and VPD did not differ between species (Table S3 available as Supplementary data at *Tree Physiology* Online). However, mean g_s_ of droughted trees measured after the break-point (from week 10) were significantly higher in *E. microcarpa* than *E. melliodora* (Table 3). Greater VPD on measurement days resulted in significant decreases in afternoon g_s_ which was uniform between species and between droughted and well-watered trees. There were no statistically significant differences in g_s_ among provenances in either species with the exception of one provenance of *E. microcarpa* (mean annual temperature [MAT] provenance) in the droughted treatment, which was slightly higher than other provenances (data not shown).

Correlations between Ψ_pd_ and climate for each provenance were compared by taking the mean Ψ_pd_ of provenances of each species averaged over weeks 10–17. Means were plotted along gradients of rainfall of the provenance home locations (Figure 4). We observed a significant linear relationship in *E. microcarpa* provenances where regions with greater median dry quarter rainfall (Figure 4a) and greater mean annual rainfall (Figure 4c) had comparatively more negative Ψ_pd_. In other words, *E. microcarpa* provenances from more arid regions experienced lower water deficits. No relationships were observed in provenances of *E. melliodora*.

**Figure 4:**
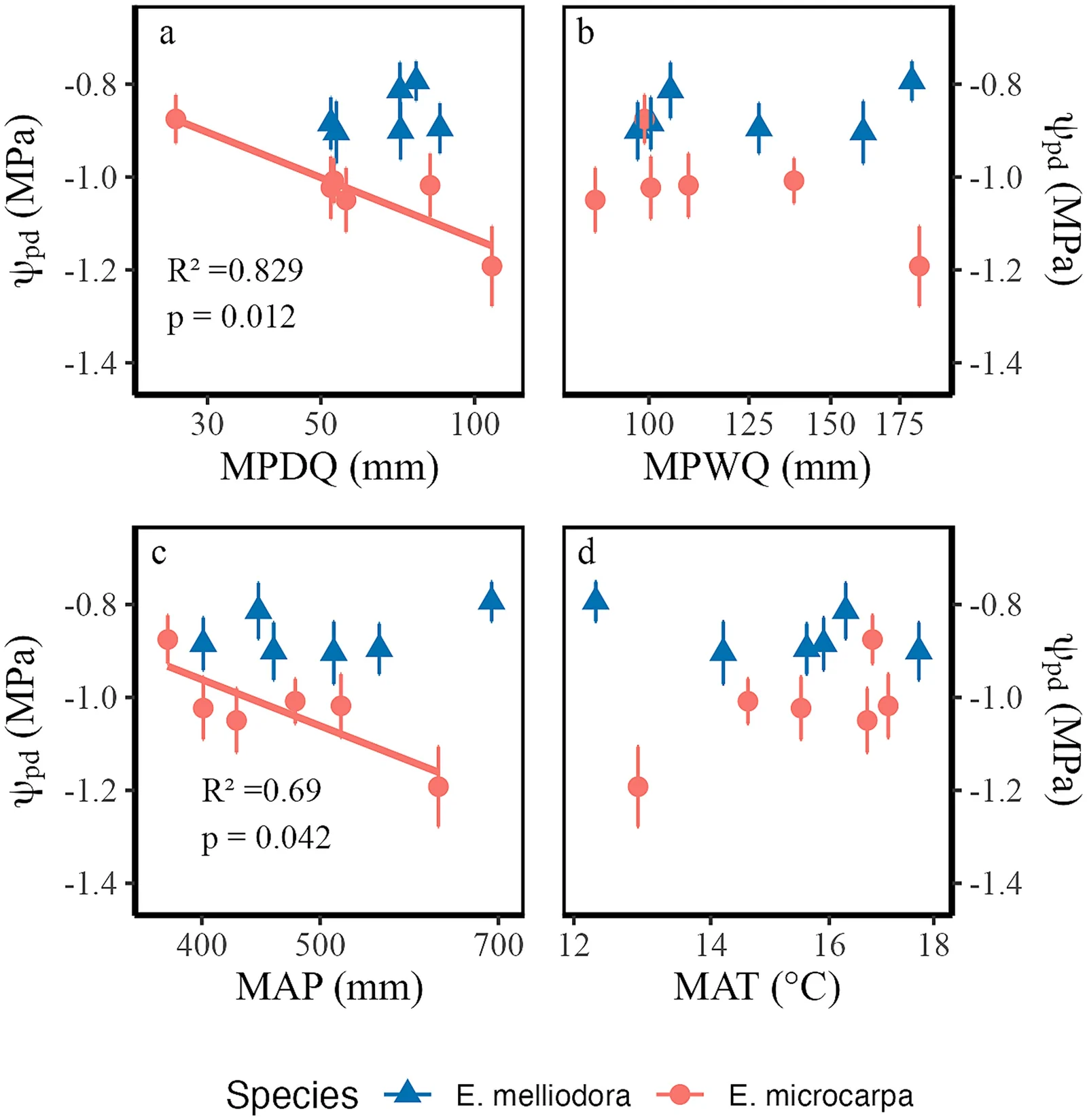
Predawn water potential (Ψ_pd_) of provenances of *E. microcarpa* (red circles) and *E. melliodora* (blue triangles) subjected to drought conditions (*n* = 4 to 6). Values are means averaged from week 10 to week 17 when treatment effects were significant. Bars indicate standard error. Means are plotted against (a) median precipitation in the driest quarter, (b) median precipitation in the wettest quarter, (c) mean annual precipitation and (d) mean annual temperature. The climate gradients on the x-axis were log transformed. A solid line shows significant (*P* < 0.05) linear relationships.

### Osmotic adjustment

*E. microcarpa* trees had significantly lower osmotic potential (π) in comparison with *E. melliodora* trees in both treatments (*P* < 0.001). The π of both species significantly decreased in both species in response to drought (π_adj_ of 0.24 MPa in droughted *E. microcarpa* and 0.20 MPa in *E. melliodora, P* < 0.001) (Figure 5). A log-linear relationship of π_adj_ and climate of origin suggests *E. microcarpa* trees from regions with greater median precipitation in the wettest quarter (MPWQ) had greater π_adj_ in comparison with *E. microcarpa* provenances from drier regions (Figure 6). No significant relationship was observed between *E. melliodora* and any climate measure (Figure 6).

**Figure 5:**
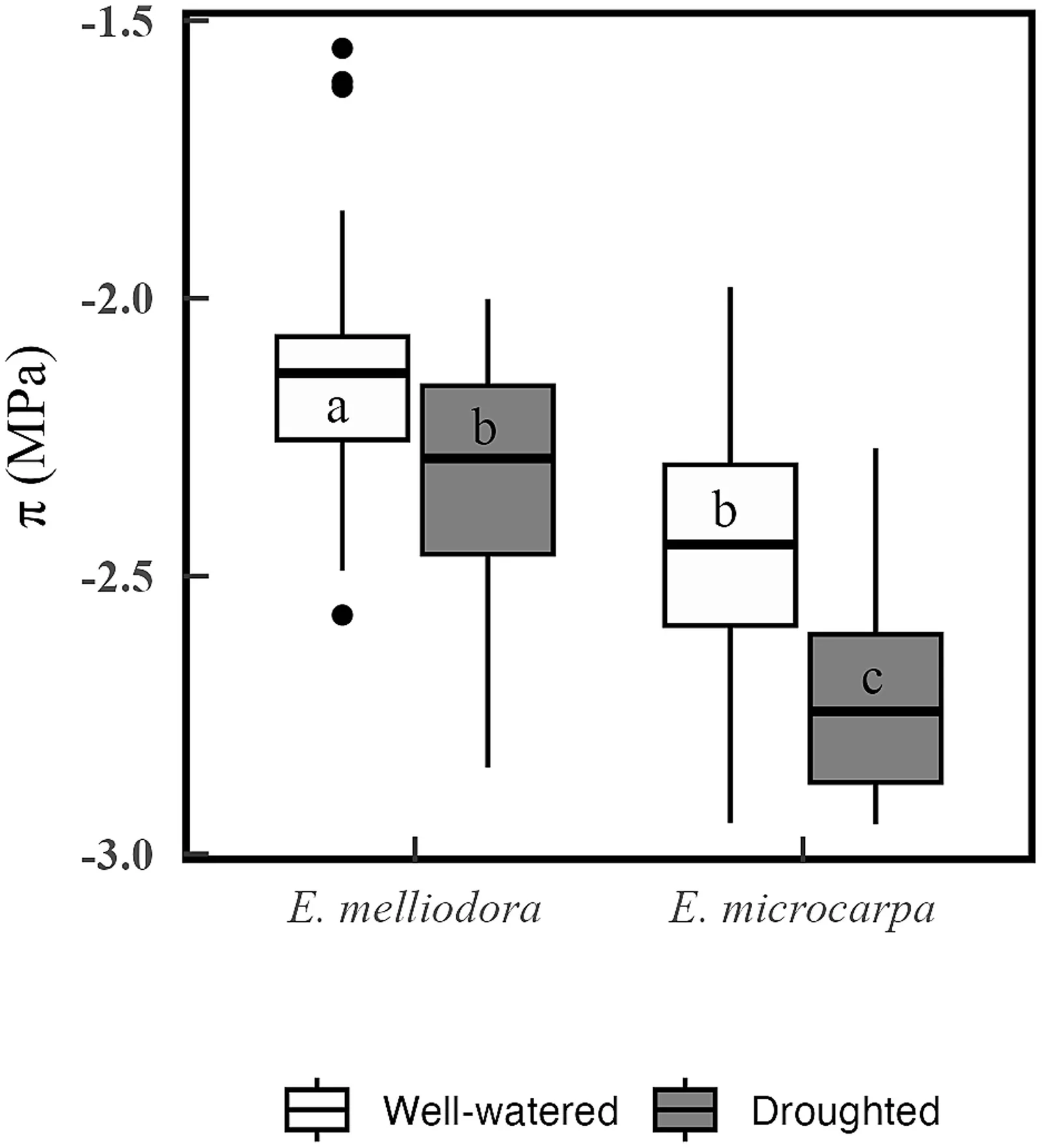
Boxplots of osmotic potential (π) of *E. melliodora* and *E. microcarpa* (*n* = 33 to 39)*.* Letters indicate significant differences observed through ANOVA.

**Figure 6:**
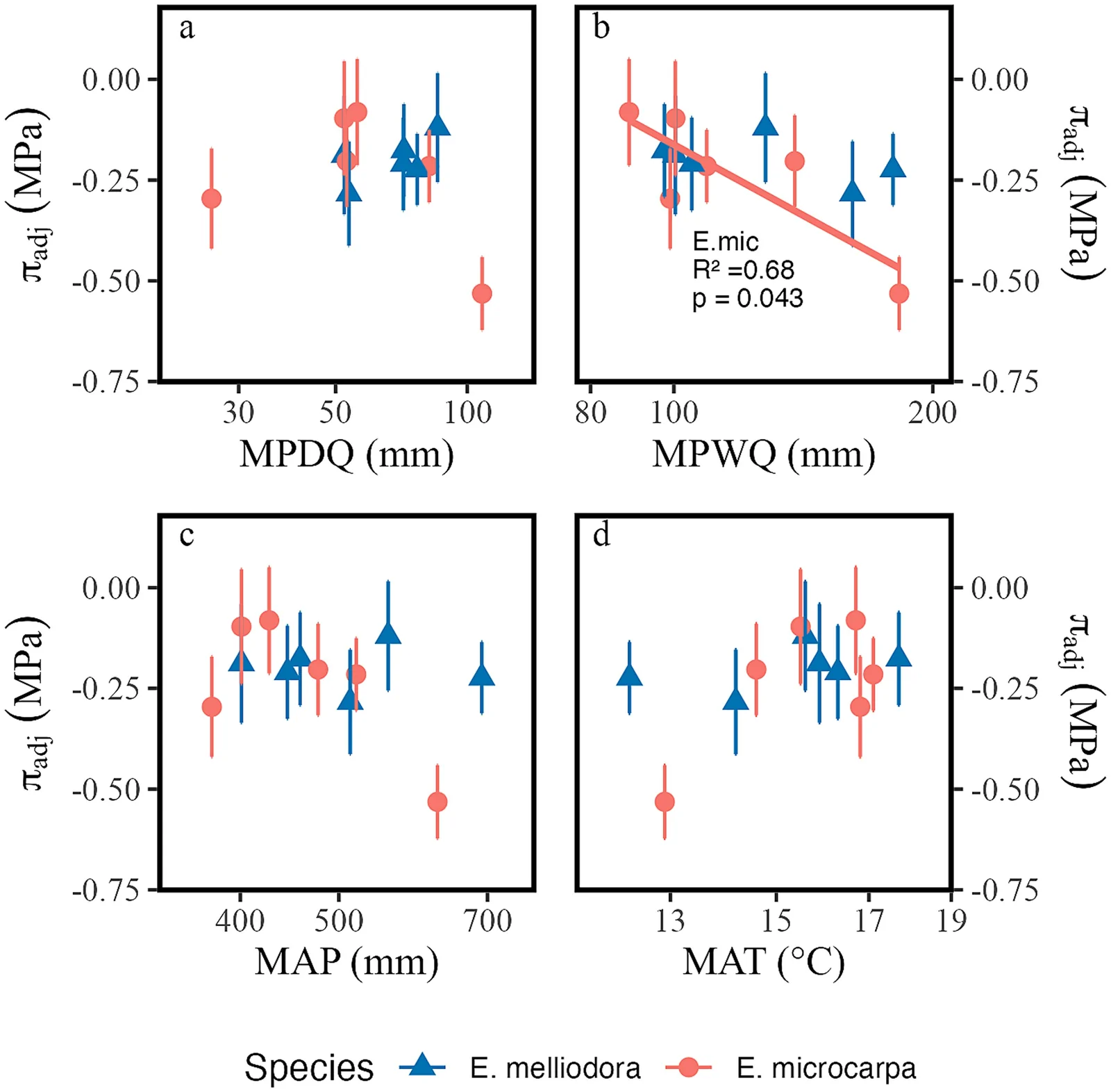
The relationship between mean provenance osmotic adjustment (π_adj_) and climate variables (*n* = 4 to 6). Circles represent *E. microcarpa* provenances and triangles represent *E. melliodora* provenances; bars indicate standard error. The climate gradients on the x-axis were log transformed. Significant linear relationships are depicted with a solid line with respective R^2^ values and *P*-values.

### Biomass, morphological traits and drought responses

Drought had a greater morphological impact on *E. microcarpa* in comparison with *E. melliodora*. Both species had comparatively lower total biomass in the drought treatment, however droughted *E. microcarpa* trees had significantly lower total biomass in comparison with droughted *E. melliodora* trees (Figure 7a and b). Differences in LA between droughted and well-watered trees was also greater in *E. microcarpa* (64%) in comparison with *E. melliodora* (42%), however the difference was not statistically significant (Figure 7c). Only *E. microcarpa* showed significant drought impacts on SLA (10% lower) (Figure 7d). Both species had a significantly lower root biomass in droughted trees, however the difference was much greater in *E. microcarpa* (Figure 7e). Both species also had significantly higher root:shoot ratios in the droughted trees in comparison with well-watered trees (Figure 7f). Tree height was similar between well-watered and droughted *E. melliodora*. In contrast, *E. microcarpa* had significantly lower tree heights in droughted trees in comparison with well-watered trees (Figure 7g). Huber value did not differ between droughted and well-watered trees in either species (Figure 7h). The only species differences in biomass and morphological traits of well-watered trees was lower SLA, lower root weight and a lower root:shoot ratio in well-watered *E. microcarpa* in comparison with *E. melliodora* trees (Figure 7d–f). In the remaining measures, the differences between species in well-watered trees were not significant.

**Figure 7:**
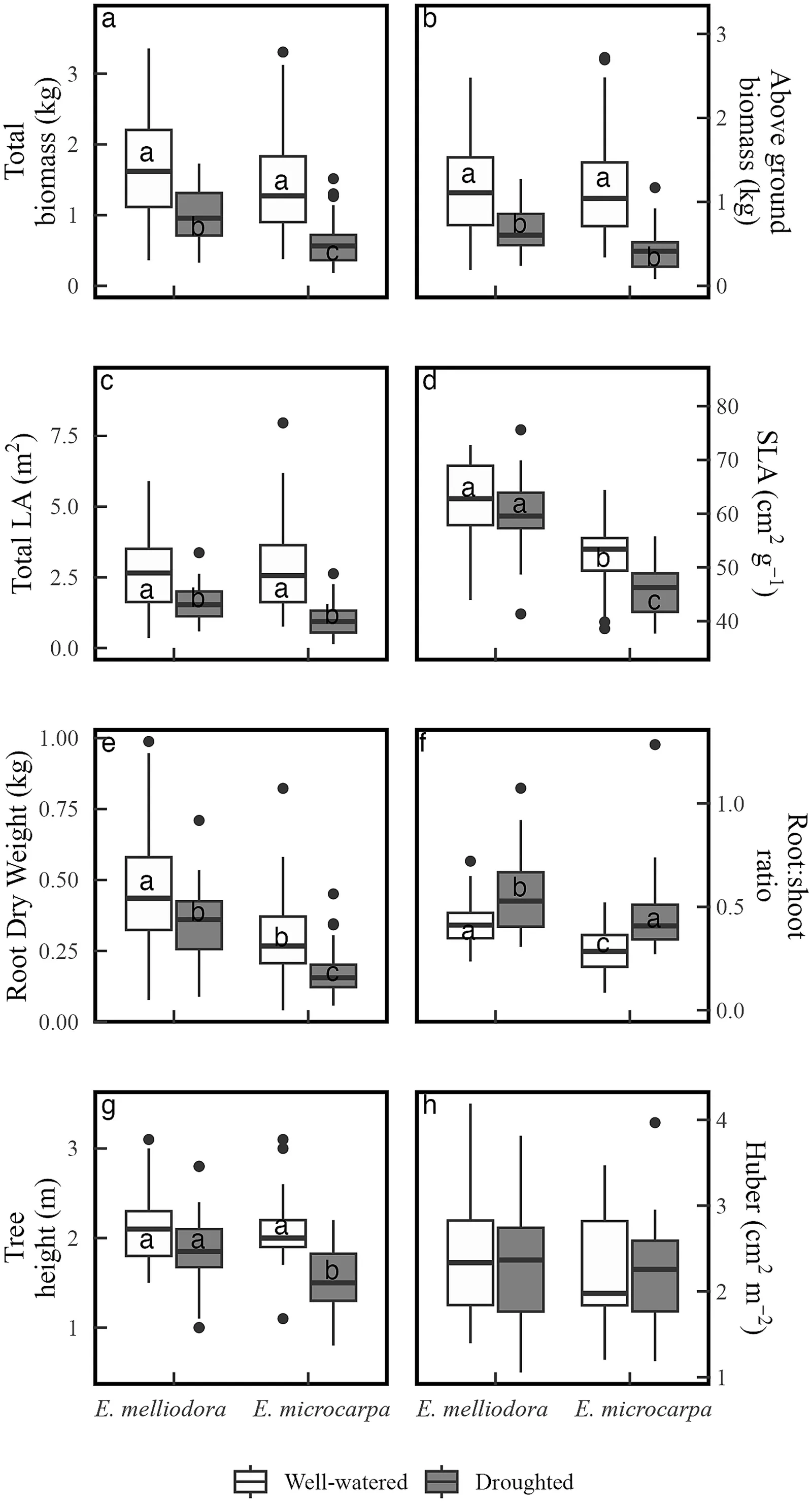
Boxplots of biomass allocation and SLA measured post-harvest (*n* = 30 to 34) of droughted (white) and well-watered (grey) trees for each species. Letters denote significant differences found through ANOVA and a subsequent Tukey post-hoc test (*P* < 0.05).

Relationships between contrasts in morphological traits between well-watered and droughted trees, and climate of origin of each provenance was limited to one relationship in *E. microcarpa*, where provenances from drier regions had comparatively lower mean heights in droughted trees, ranging from 0.9 m lower in the driest provenance to 0.4 m lower in the wettest provenance (Figure 8e and g).

**Figure 8:**
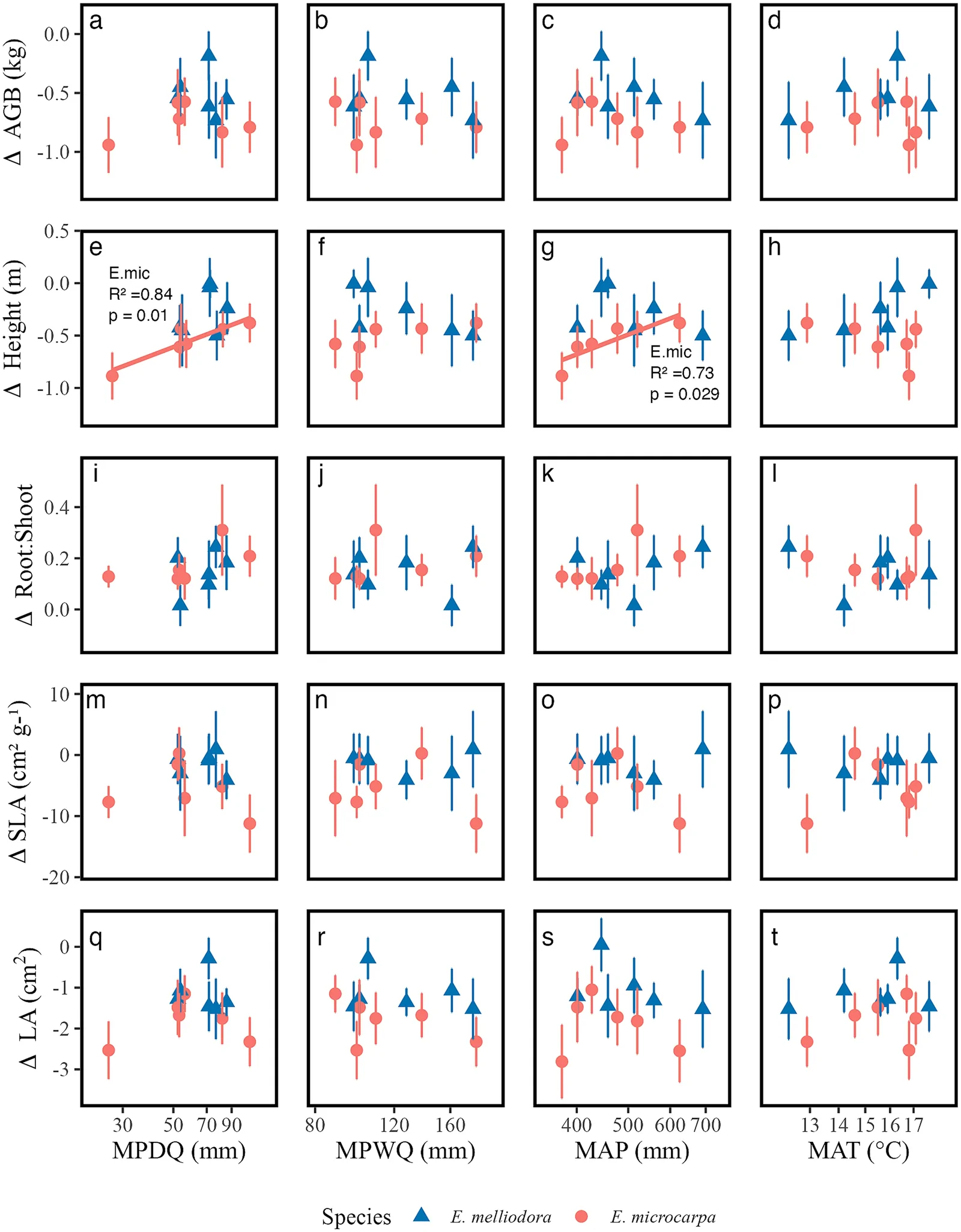
Comparative differences of morphological traits between droughted and well-watered trees (y-axis) and climate of origin (x-axis) for six provenances of *E. microcarpa* (red circles) and *E. melliodora* (blue triangles) (*n* = 4–6), bars indicate standard error. Values are mean differences between droughted trees in comparison with well-watered trees. All measurements were performed after the completion of the 17-week drought trial. The climate gradients on the x-axis were log transformed. Correlations were tested via linear models and relationships with significance of *P* < 0.05 are presented with a line and respective R^2^ values.

## Discussion

We hypothesized that *E. microcarpa* would have greater tolerance to drought and subsequently greater growth and survival during drought in comparison with *E. melliodora*. We also hypothesized that within each species, provenances from drier regions would exhibit greater tolerances to drought. Our results only partially support the first hypothesis. While the drought treatment was not substantial enough to result in mortality in any of the trees, we observed different responses between the species. *E. microcarpa* exhibited traits associated with greater tolerances to drought which coincided with significantly reduced growth and marginally greater water deficits. We attribute the growth reduction in *E. microcarpa* to increased plasticity in response to drought, as opposed to direct impacts of physiological function due to water deficits. There was limited support for the second hypothesis as there was little evidence of varying adaptation between provenances in *E. microcarpa*, and no evidence of adaptive differences in provenances of *E. melliodora*.

### Water-use strategies

The drought treatment resulted in decreases of Ψ in both species. However, Ψ did not reach levels of critical stress for either species during the 17-week experiment, despite trees receiving no irrigation for this period (Figure 2a). Mean Ψ_pd_ of droughted *E. microcarpa* was −1.26 MPa in week 17 which was higher than the Ψ_pd_ of −4.0 MPa previously observed in field experiments in this species (Myers and Neales 1984). The Ψ_md_ was consistently lower than Ψ_pd_ over sequential weeks, and we can infer that both species subjected to drought were still able to transpire and sufficiently rehydrate leaves overnight. Both species also maintained Ψ_md_ above Ψ_tlp_ despite a gradual decrease in Ψ_pd_. This was driven in part by stringent stomatal regulation in response to both Ψ and VPD (Figure 3b and c) that is evident in many *Eucalyptus* species (White et al. 2000, Li et al. 2019). Furthermore, both species showed considerable root development, likely allowing the trees to access sufficient soil moisture to partially rehydrate overnight. Although the drought treatment resulted in decreases in Ψ_pd_ and Ψ_md_ in both species, the water deficit was comparatively greater in *E. microcarpa.* A comparatively lower Ψ_pd_ in droughted *E. microcarpa* may suggest that this species was under more drought stress, despite the difference only being small.

*Eucalyptus microcarpa* had consistently higher g_s_ in comparison with *E. melliodora* throughout the experiment in both well-watered and droughted trees (Figure 3b) and this led to more negative Ψ_md_ in droughted *E. microcarpa*. Therefore, despite exhibiting more negative Ψ_pd_, *E. microcarpa* still maintained higher relative g_s_, suggesting that *E. microcarpa* is more tolerant to low Ψ than *E. melliodora*. Both species had a significant reduction in g_s_ when VPD was above 4 kPa (Figure 3d). However, we were unable to ascertain an accurate measure of stomatal closure that may have been caused by drought, as both species appeared to successfully avoid drought to some degree. Interestingly, we did observe higher g_s_ in well-watered *E. microcarpa* in comparison with well-watered *E. melliodora* which did not result in differences in Ψ_md_ between the species (Figure 3c). This could indicate either greater hydraulic conductance or greater hydraulic capacitance in *E. microcarpa* trees. *Eucalyptus* species originating from arid regions tend to have narrower xylem vessels, resulting in a reduction in maximum hydraulic conductance (Pfautsch et al. 2016), while hydraulic capacitance has been shown to be an important trait in extending eucalypt survival during water stress (Gleason et al. 2014).

Osmotic potential of expressed sap (π) also varied between the two species in well-watered and droughted treatments (Figure 5c). In both cases, *E. microcarpa* had more negative π than *E. melliodora*. π is highly correlated with the water potential at turgor loss point (Bartlett et al. 2012), and is considered a key indicator of tolerance to low water availability. A study of osmotic potential in six species of *Eucalyptus* species showed lower π in species from drier regions (Merchant et al. 2007). This is congruent with the findings of this study, as we observed lower π in *E. microcarpa*. However, both species had similar levels of osmotic adjustment despite species differences in π in well-watered trees, and Ψ_pd_ in droughted trees.

### Biomass allocation and morphological plasticity

Both species showed similar morphological structure in well-watered conditions, with small differences in root:shoot ratios and SLA. Typically, species from comparatively drier regions have higher root:shoot ratios (Mokany et al. 2006), however the opposite was observed in the present study given that *E. melliodora* had a greater root:shoot ratio, though the differences observed were small. The SLA was significantly greater in well-watered *E. melliodora* in comparison with *E. microcarpa*, which is congruent with expectations, as SLA is typically greater in species from regions with higher rainfall (Schulze et al. 1998). All other morphological traits of the two species were similar in well-watered conditions. However, the drought treatment resulted in significantly greater changes in *E. microcarpa* in comparison with *E. melliodora*. The lack of plasticity observed in *E. melliodora* may be because plasticity was not induced as the trees were not subjected to sufficient water deficits. The inherent greater allocation of resources to root growth may have allowed this species to largely avoid drought stress. In contrast, when *E. microcarpa* trees were subjected to a water deficit, there was a significantly greater reduction in tree height, above ground biomass, total LA and SLA, as well as a significantly greater increase in root:shoot ratios. Increases in root:shoot ratio have been observed in drought stressed *Eucalyptus microtheca* trees (Li et al. 2000) and *Eucalyptus globulus* (Madeira et al. 2002). A reduction in SLA in response to drought has also been well described in *Eucalyptus* species and shows a strong association with aridity (Pita and Pardos 2001, Anderegg et al. 2021).

A reduction in tree height and biomass in water-limited trees is intuitive, however the variation between the species gives rise to questions regarding the potential bidirectional relationship between growth responses and drought stress: what constitutes a ‘drought response’ and a ‘drought effect’? Both species showed only negligible water deficits as evidenced by Ψ_pd_ at week 17 of the drought treatment, averaging −1.26 MPa in *E. microcarpa* and −1.06 MPa in *E. melliodora*. However, height and growth reductions were much greater in *E. microcarpa* in comparison with *E. melliodora*. It is difficult to discern whether this level of water deficit resulting in growth reductions in *E. microcarpa* is a passive constraint on growth (the water deficit prevents cell function and replication) or whether this is a regulated response (water deficit is driving hormonal responses that result in growth reductions and different biomass allocations). It is perhaps more likely that a reallocation of resources to leaves (decreases in SLA), and significant investment in osmotic adjustment has resulted in decreases in overall biomass and height in *E. microcarpa*. This would suggest that the growth reductions observed in this study are more likely to be regulated responses rather than direct, systemic impacts of water deficits—the ‘safe and slow’ theory of tree adaptation to drought (Oliveira et al. 2021, Guillemot et al. 2022). In contrast to the substantial plasticity of *E. microcarpa*, was a lack of plasticity in *E. melliodora*. Though *E. melliodora* did have marginally less hydraulic stress as evidenced by less negative water potentials, there are questions as to whether the stress was not sufficient to induce plasticity, or whether plastic responses to water deficits are less pronounced in this species.

### Provenance-driven differences in plasticity

The plasticity of *E. microcarpa* in response to the water deficit was not uniform within the species, as the variation of some morphological responses appeared to show associations with rainfall of origin. This is of particular interest, as provenance variation appeared to have no association with climate under well-watered conditions in *E. microcarpa*. Therefore, there may be location-specific adaptation differences in plastic responses to water deficits in *E. microcarpa*. In contrast, no association between provenance rainfall and morphological plasticity was observed in *E. melliodora.* This is less surprising, as plasticity in response to the drought treatment in this species was limited in general. Furthermore, a previous study of *E. melliodora* found no evidence of site-specific variations in phenotypic plasticity (Supple et al. 2018).

Intraspecific variation in plasticity has been well documented in studies of both flora and fauna (Kelly 2019). In a reciprocal transplant study of *Populus fremontii*, populations from hotter regions exhibited four times greater phenological plasticity in comparison with cooler populations (Cooper et al. 2019). In *Pinus ponderosa*, positive relationships between summer heat-moisture index of populations and their respective phenotypic plasticity were observed (De La Mata et al. 2022). Furthermore, a systematic literature review shows provenance differences of European tree species in many traits, including growth (Aspalter et al. 2025). In eucalypts, a common garden provenance study showed *Eucalyptus tricarpa* had greater plasticity of leaf thickness in trees from drier regions, yet greater plasticity in leaf size in trees from wetter regions (Mclean et al. 2014). Studies on *E. microcarpa* have shown significant genome-wide diversity within the species (Jordan et al. 2016) as well as intraspecific variation of traits related to drought adaptation (Jordan et al. 2020). In this study, *E. microcarpa* provenances from drier regions exhibited less water deficit, less osmotic adjustment, as well as differences in drought responses including a relatively larger reduction in tree height. There are well-known associations between water availability and tree height in *Eucalyptus* species found along rainfall gradients in Australia (Givnish et al. 2014). In evolutionary terms, tree height is largely driven by competition (King 1990), and competition for light decreases in low-productivity forests where water becomes a significant limiting factor (Prior and Bowman 2014). Therefore, small differences in provenance growth strategies, or ‘trade-offs’, may be present in *E. microcarpa*; trees from drier regions reduce height in water-limiting environments where competition for light is less important. In contrast to this, *E. microcarpa* provenances from wetter regions are more likely to face competition from other species in mixed forests as water becomes less of a limiting factor in that ecosystem. Populations from these regions may therefore have a high-risk strategy and reach lower Ψ to maintain tree height to ensure competitiveness in more productive forests.

### Implications for land restoration

This study shows that two closely related species of *Eucalyptus* that occupy overlapping niches exhibit significantly different strategies when subjected to water limitations. *Eucalyptus melliodora* avoided stress by significantly increasing resource allocation to root biomass, which allowed this species to have comparatively greater control over internal water deficits and maintain comparatively greater growth. In contrast to this, *E. microcarpa* had significant reductions in tree growth, likely exacerbated by limited root growth and subsequently greater water deficits. While both the climate distribution data and functional trait expression suggest that *E. microcarpa* is a more drought-adapted tree, this species was more impacted by the water deficit in this experiment. This suggests that neither climate envelope data nor functional trait expression per se are good predictors for species ‘performance’ under certain levels of drought stress, as *E. melliodora*, the ‘wetter’ species, had better, i.e. less drought impacted performance in this experiment. However, the significant plasticity in leaf traits, osmotic adjustment and growth reduction in *E. microcarpa* may present an advantage in a more severe drought than what was applied in this study.

When considering provenance adaptation, this present study shows the importance of understanding plasticity in respect to intraspecific variation. The only associations observed in this study involved *E. microcarpa* subjected to the water deficit. Therefore, provenance-driven variation in drought adaptation was revealed by drought stress and subsequent plastic responses. Some of these variations in plasticity, namely plasticity in tree heights, may also have implications in the context of forest restoration. If drought-adapted genotypes exhibit significant height reductions in response to moderate water limitation, this may impact that genotype’s ability to compete in a forest ecosystem. While a reduction in tree height and an ability to maintain relatively less negative Ψ_pd_ may result in persistence in severe droughts (Stovall et al. 2019), in moderate droughts the impacts of resource competition may result in these putatively adapted genotypes being relatively worse off. Furthermore, when considering the monitoring of restored forest landscapes, common metrics such as tree diameter and height are often utilized to determine the performance of an individual tree (Niklas 1993). However, there may be issues with conflating the performance of a genotype in a landscape, even one undergoing moderate drought impacts, with said genotype exhibiting an adaptive advantage to drought. The genotypes that may have the greatest ability to persist in severe droughts are the same genotypes that may appear to be worse off in moderate drought conditions.

## Conclusion

This study investigated drought responses in provenances of two closely related *Eucalyptus* species. Both species subjected to the drought treatment endured only mild water deficits, however the deficits were greater in *E. microcarpa*, the species we assumed to be better adapted to drought. We suggest that this is due to a mixture of an inherent greater allocation to root biomass of *E. melliodora* and a heightened plastic response to water deficits in *E. microcarpa*. The root growth in *E. melliodora* likely enabled this species to access more water during the drought period and subsequently reduce water deficits. In contrast, *E. microcarpa* allocated less biomass to root growth and subsequently endured marginally greater water deficits. However, this coincided with considerable plastic responses of morphology in *E. microcarpa* that were not observed in *E. melliodora*. Within-species provenance differences were limited and only observed in *E. microcarpa* trees subjected to drought, suggesting that plasticity may differ with climate of origin in this species. Provenances from regions with reduced rainfall showed the least stress via less negative Ψ_pd_ and less osmotic adjustment. We suggest this is driven by an increased plastic response resulting in a greater reduction in tree height and growth. This has three main implications: genotypes that may be adapted to significant drought events could potentially be outcompeted in less severe drought due to heightened plastic responses and subsequent reductions in tree height. Secondly, assessing the health and success of a provenance trial via metrics such as tree height and growth factors may be misleading, if reductions in tree height are associated with adaptation to drought. Thirdly, if provenance adaptation in eucalypts is primarily a result of differences in plasticity, then subjecting provenances to significant drought stress may be integral to developing an understanding of the potential of intraspecific variation in adaptation to drought.

## Supplementary Material

Browning_R_Tree_Phys_Supp_July_2026_tpag117

## Data Availability

The authors confirm that the data collected in this experiment is available upon reasonable request.
