# Supplementary material for: Physiological responses and morphological adjustments in provenances of two woodland species in a chronic drought experiment
Source: Tree Physiol. 2026 Aug 22;46(9):tpag117. doi: 10.1093/treephys/tpag117 (PMC13618798; doi:10.1093/treephys/tpag117)
Supplement: Supplementary file 1 [file browning_r_tree_phys_supp_july_2026_tpag117.docx]

Supplementary Table 1: Weather station data collected from bom.gov.au.

| CON | Site name:  CONDOBOLIN AG RESEARCH STN | DEN | Site name:  DENILIQUIN (VISITOR INFORMATION CENTRE) |
| --- | --- | --- | --- |
|  | **Site number:**  050052 |  | **Site number:**  074128 |
|  | **Latitude:**  33.07 °S   **Longitude:**  147.23 °E |  | **Latitude:**  35.53 °S   **Longitude:**  144.97 °E |
|  | **Elevation:**  195 m |  | **Elevation:**  96 m |
|  | **Commenced:**  1954  **Status:** Open |  | **Commenced:**  1858  **Status:** Open |
| JUN | **Site name:**  JUNEE TREATMENT WORKS | **LG** | **Site name:**  ARARAT PRISON |
|  | **Site number:**  073019 |  | **Site number:**  089085 |
|  | **Latitude:**  34.85 °S   **Longitude:**  147.57 °E |  | **Latitude:**  37.28 °S   **Longitude:**  142.98 °E |
|  | **Elevation:**  280 m |  | **Elevation:**  295 m |
|  | **Commenced:**  1891  **Status:** Open |  | **Commenced:**  1969  **Status:** Open |
| NAR | **Site name:**  NARRANDERA AIRPORT AWS | **SAR** | **Site name:**  ST ARNAUD |
|  | **Site number:**  074148 |  | **Site number:**  079040 |
|  | **Latitude:**  34.71 °S   **Longitude:**  146.51 °E |  | **Latitude:**  36.62 °S   **Longitude:**  143.26 °E |
|  | **Elevation:**  145 m |  | **Elevation:**  240 m |
|  | **Commenced:**  1967  **Status:** Open |  | **Commenced:**  1880  **Status:** Open |
| BAC | Site name:  LAVERTON RAAF | FIF | Site name:  TULLAMORE (KITCHENER ST) |
|  | **Site number:**  087031 |  | **Site number:**  050037 |
|  | **Latitude:**  37.86 °S   **Longitude:**  144.76 °E |  | **Latitude:**  32.63 °S   **Longitude:**  147.57 °E |
|  | **Elevation:**  20 m |  | **Elevation:**  244 m |
|  | **Commenced:**  1941  **Status:** Open |  | **Commenced:**  1914  **Status:** Open |
| GRI | **Site name:**  GRIFFITH AIRPORT AWS | **MAT** | **Site name:**  DENILIQUIN (VISITOR INFORMATION CENTRE) |
|  | **Site number:**  075041 |  | **Site number:**  074128 |
|  | **Latitude:**  34.25 °S   **Longitude:**  146.07 °E |  | **Latitude:**  35.53 °S   **Longitude:**  144.97 °E |
|  | **Elevation:**  134 m |  | **Elevation:**  96 m |
|  | **Commenced:**  1960  **Status:** Open |  | **Commenced:**  1858  **Status:** Open |
| QUO | **Site name:**  QUORN | **WED** | **Site name:** WEDDERBURN (POST OFFICE) |
|  | **Site number:**  19038 |  | **Site number:** 080061 |
|  | **Latitude:**  32.35 °S   **Longitude:**  138.04 °E |  | **Latitude:**  36.42° S **Longitude:  143.61° E** |
|  | **Elevation:**  295 m |  | **Elevation:**  200 m |
|  | **Commenced:**  1881  **Status:** Open |  | **Commenced:** 1882  **Status:** Open |

Supplementary Table 2: Model comparison of response of Ψ_md_ and g_s_ for all weeks.

Maximum Likelihood (ML) models were used for AIC comparisons.

| Response | Species | Model | df | AIC |
| --- | --- | --- | --- | --- |
| Ψ_md_ | *E. melliodora* | Week only | 8 | 1659.6809 |
|  |  | VPD only | 8 | 1016.0778 |
|  |  | VPD + Week | 12 | 793.2131 |
|  | *E. microcarpa* | Week only | 8 | 1875.9648 |
|  |  | VPD only | 8 | 1103.4641 |
|  |  | VPD + Week | 12 | 776.3809 |
| g_s_ | *E. melliodora* | Week only | 8 | 3947.8 |
|  |  | VPD only | 8 | 3934.92 |
|  |  | VPD + Week | 12 | 3115.12 |
|  | *E. microcarpa* | Week only | 8 | 4453.087 |
|  |  | VPD only | 8 | 4641.05 |
|  |  | VPD + Week | 12 | 4211.621 |

Supplementary Table 3: Models comparisons between-species in droughted trees. Null model is of a single, shared smooth. Full models include separate smooths for each species. X^2^ and df are derived from likelihood ratio tests of nested models.

| Response | Model | df | AIC | Log-likelihood | X^2^ | df (X^2^) | p |
| --- | --- | --- | --- | --- | --- | --- | --- |
| Ψ_pd_ | Null (shared smooth) | 5 | 206.78 | -98.39 | - | - |  |
|  | Full (species smooths) | 8 | 199.06 | -91.53 | 13.72 | 3 | 0.003 |
| Ψ_md_ | Null (shared smooth) | 7 | 1007.67 | -496.83 | - | - |  |
|  | Full (species smooths) | 12 | 1024.41 | -500.2 | 6.74 | 5 | 0.241 |
| g_s_ | Null (shared smooth) | 7 | 4357.86 | -2171.93 | - | - |  |
|  | Full (species smooths) | 12 | 4360.85 | -2168.43 | 7.01 | 5 | 0.220 |

Supplementary Table 4: Model comparison of provenance differences of droughted trees within each species. Null models are comprised of a single, shared smooth term. Full models are comprised of provenance-specific smooths and a parametric provenance term.

| Species | Response Variable | Model | df | AIC |
| --- | --- | --- | --- | --- |
| *E. microcarpa* | Ψ_pd_ | Null | 5 | 97.87 |
|  |  | Full | 20 | 92.79 |
|  | Ψ_md_ | Null | 7 | 514.18 |
|  |  | Full | 32 | 648.36 |
|  | g_s_ | Null | 7 | 2087.76 |
|  |  | Full | 32 | 2123.68 |
| *E. melliodora* | Ψ_pd_ | Null | 5 | 104.84 |
|  |  | Full | 20 | 122.58 |
|  | Ψ_md_ | Null | 7 | 511.59 |
|  |  | Full | 32 | 622.35 |
|  | g_s_ | Null | 7 | 2266.63 |
|  |  | Full | 32 | 2294.51 |

N

Supplementary Figure 1. A diagram depicting the layout of the trees in the rainout shelter. The area shaded blue is the area covered by the rainout shelter. Trees were planted along the border to remove edge effects (not shown in diagram). The areas shaded green show each plot where trees were planted. The two rows that were excluded are highlighted in orange.
